# Terahertz Imaging and Spectroscopy in Cancer Diagnostics: A Technical Review

**DOI:** 10.34133/2020/2547609

**Published:** 2020-09-25

**Authors:** Yan Peng, Chenjun Shi, Xu Wu, Yiming Zhu, Songlin Zhuang

**Affiliations:** Terahertz Technology Innovation Research Institute, Shanghai Key Lab of Modern Optical System, Terahertz Science Cooperative Innovation Center, University of Shanghai for Science and Technology, Shanghai Institute of Intelligent Science and Technology, Tongji University, Shanghai, China

## Abstract

Terahertz (THz) waves are electromagnetic waves with frequency in the range from 0.1 to 10 THz. THz waves have great potential in the biomedical field, especially in cancer diagnosis, because they exhibit low ionization energy and can be used to discern most biomolecules based on their spectral fingerprints. In this paper, we review the recent progress in two applications of THz waves in cancer diagnosis: imaging and spectroscopy. THz imaging is expected to help researchers and doctors attain a direct intuitive understanding of a cancerous area. THz spectroscopy is an efficient tool for component analysis of tissue samples to identify cancer biomarkers. Additionally, the advantages and disadvantages of the developed technologies for cancer diagnosis are discussed. Furthermore, auxiliary techniques that have been used to enhance the spectral signal-to-noise ratio (SNR) are also reviewed.

## 1. Introduction

**Figure 1:**
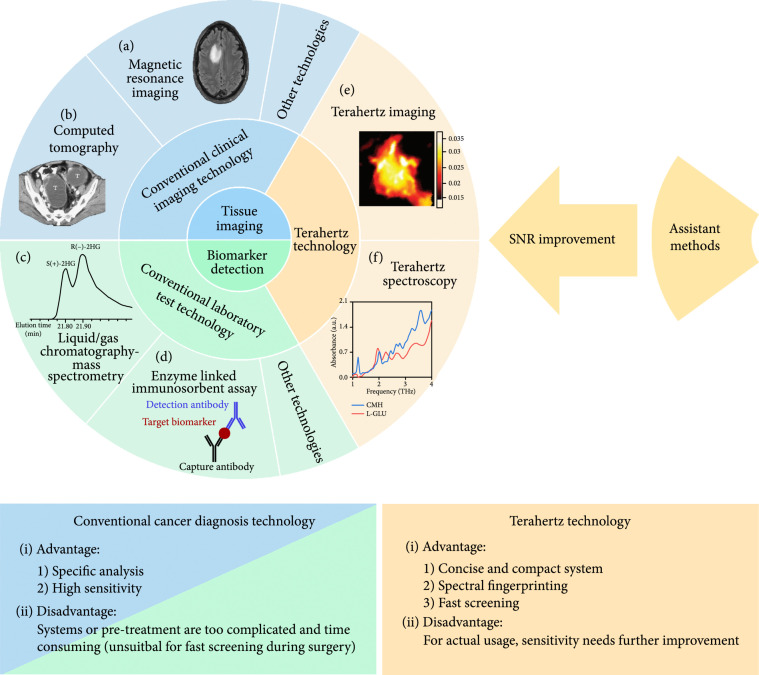
Multiple cancer diagnosis methods. (a) Magnetic resonance imaging of a brain tumor (from Ref. [2]). (b) Computed tomography scan of bilateral serous cystadenocarcinomas in a 50-year-old woman with contrast enhancement shows bilateral ovoid tumors (T) with septa and mural nodules (from Ref. [3]). (c) Basic principle of the enzyme-linked immunosorbent assay: the capture antibody captures the target biomarker then binds to the detection antibody. (d) Separation of the (R) and (S) enantiomers of the cancer biomarker 2HG by liquid chromatography-mass spectrometry (from Ref. [4]). (e) Terahertz imaging of a breast tumor (from Ref. [5]). (f) Terahertz absorption spectra of creatine (red line) and L-Glutamic acid (blue line).

The early diagnosis of cancer is very important for patient treatment and recovery. If cancers can be confirmed and classified at an early stage, the patient survival rate can be greatly improved [1]. As shown in Figure 1, current clinical cancer diagnostic techniques can be divided into two catalogs: tissue imaging and spectroscopic biomarker detection.

Conventional imaging technologies, including magnetic resonance imaging (MRI), computed tomography (CT), and ultrasound (US), can be used to detect tissue structural differences, such as heterogeneity, cell apoptosis, and cell density, that are related to cancer [6]. Laboratory assays based on chromosomal analysis, methylation analysis, proteomic tools, immunological reactions, or antibody reactions [7] can be used to detect biomarkers (*e.g.*, DNA or proteins) that are associated with cancer. However, to achieve high sensitivity and accuracy, these techniques mostly require large devices or complicated preprocessing steps.

Terahertz (THz) waves are electromagnetic waves with frequency in the range from 0.1 to 10 THz [8]. As THz waves are located between the microwave and infrared regions on the electromagnetic spectrum, they exhibit features of both regions: they are nonionizing, noninvasive, and phase-sensitive to polar substances, can be used for spectral fingerprinting, and coherent detection, and offer good resolution (up to 50 *μ*m) and penetration capabilities [9]. Based on these unique features, THz waves have potential applications in many fields [10-15]. In oncology, the nonionizing and noninvasive nature and good penetration capabilities of THz waves make them suitable to inspect tissue both *in vivo* and *ex vivo* with high accuracy while causing minimal harm. Furthermore, spectral fingerprinting with THz waves can enable qualitative and quantitative analysis of cancer biomarkers.

THz spectroscopy is compared with other types of electromagnetic spectroscopy in terms of the information they can obtain in Table 1 [16-19]. THz spectroscopy detects the collective behavior (vibration and rotation) of molecules. Therefore, THz spectroscopy can be used to differentiate substances with different molecular structures as well as polymorph and chiral substances, even those that have the same elements and molecular bonds [20]. Although microwave spectroscopy can also detect molecular rotation in such substances, its wavelength is longer than that of THz wave spectroscopy, resulting in a relatively low resolution.

**Table 1:** Information detected by different electromagnetic spectroscopies.

Spectroscopy type	Detectable information
Energy-dispersive X-ray spectroscopy	Elemental composition
Microwave spectroscopy	Rotation of molecules
Raman/infrared spectroscopy	Fundamental vibration of chemical bonds
Ultraviolet-visible light (UV/VIS) spectroscopy	Electron excitation of specific molecules
Terahertz spectroscopy	Collective behavior of molecules (vibration and rotation)

Given the above advantages of THz technologies and considering that they are generally compact systems, THz technologies have the potential to be applied for rapid tissue imaging during surgery. For cancer tissue imaging, THz imaging can distinguish cancerous and peritumoral tissue from normal tissue with clear boundaries, thus providing more intuitive information to the surgeon to aid in the removal of the cancerous and peritumoral tissue. However, the penetration depth of THz waves in fresh tissue is limited to the water absorption depth (about 276 *μ*m for cancer tissue at 0.5 THz) [21, 22]. Therefore, current studies of THz imaging for cancer mainly focus on imaging excised tissue or using reflection imaging systems to study the surface layer of tissue, both of which can reduced the influence of water absorption. Furthermore, current studies of THz imaging are based mainly on differences in water content, which can only be used as a reference instead of a specific identification [Reference].

Biomarker detection can be helpful in the diagnosis of cancer. As different substances have different THz spectra (*i.e.*, spectral “fingerprints”), THz spectroscopy can be applied to realize fast and accurate identification of biomarkers in cancer tissue. Furthermore, THz spectroscopy can be combined with various algorithms to realize the quantitative analysis of cancer biomarkers, which may be a potential tool for rapid cancer staging. However, in most cases, various substances (such as water, proteins, fat, fiber, and other organic components) are also present in the tissue, and the biomarker concentration is usually very low. These conditions result in a low spectral SNR, which gives rise to absorption peaks that cannot be readily identified [23]. Therefore, current THz-spectroscopy-based studies of biomarkers are mostly done on pure biomarkers. If we can construct THz images based on the resonance peaks of biomarkers, cancerous areas can be identified more accurately. However, further development is required to realize this objective as current imaging systems with high-power continuous-wave THz sources can only generate THz waves at a fixed frequency, and pulsed THz sources cannot provide enough spectral power. Thus, both THz imaging and spectroscopy are limited by source energy, leading to poor SNR in the spectral results. To address this issue, some researchers are working to enhance the spectral SNR, which is expected to help detect biomarkers in mixed samples.

In this paper, we summarize recent studies of THz imaging and spectroscopy in cancer diagnosis from the past 5 years. We also present auxiliary methods to improve the SNR or enhance the THz signal. These works demonstrate the promise of using THz technologies for cancer diagnosis.

## 2. Terahertz Imaging in Cancer Diagnosis

Given the molecular fingerprinting and nonionizing features of THz waves, THz imaging can realize accurate and safe tissue imaging. However, because THz waves are readily absorbed by water, cancer tissue is generally studied *ex vivo* (*e.g.*, as freshly excised tissue or paraffin-embedded tissue) after cutting into slices that THz waves can penetrate. Table 2 shows the *ex vivo* THz imaging studies that have been conducted during the past 5 years. The resolution of THz imaging is improving with recent research advancements, particularly the development of near-field imaging methods.

**Table 2:** Current *ex vivo* THz imaging studies.

Authors	THz system	Imaging target	Results
Martin et al., 2016 [24]	A continuous-wave THz imaging system working at 0.584 THz with circular polarization	Fresh tumor and normal human skin tissue specimens	Contrast between cancerous and normal tissues was found with a resolution of 0.15 mm
Bowman et al., 2016 [25]	A pulsed THz imaging and spectroscopy system	Excised paraffin-embedded breast tissue with breast invasive ductal carcinoma	The carcinoma areas exhibited lower transmission and higher reflection than normal areas as defined based on pathology
Yamaguchi et al., 2016 [26]	A reflection THz time-domain spectroscopy system	Fresh and paraffin-embedded tissues from a rat glioma model	A difference of 0.02 (0.8-1.5 THz) in the refractive index was found between glioma and normal area
Wahaia et al., 2016 [27]	A continuous-wave THz imaging system working at 0.59 THz	Dehydrated human colon tissues	The imaging resolution reached 500 *μ*m
Grootendorst et al., 2017 [28]	A handheld THz pulsed imaging system	Freshly excised breast cancer samples	The identification accuracy of cancerous areas reached 75%
Bowman et al., 2018 [29]	A pulsed THz imaging and spectroscopy system	Freshly excised murine xenograft breast cancer tumors	Comparison with pathology results showed an accuracy above 80%
Cassar et al., 2018 [30]	A pulsed THz imaging system with a reflection mode	Freshly excised breast tissue	The spatial resolution reached 1 mm
Vohra et al., 2018 [31]	A pulsed THz imaging system with a reflection mode	Freshly excised and formalin/paraffin-fixed breast tumor tissues from a mouse model	Cancerous areas exhibited the highest reflection and agreed with the pathology results
Yeo et al., 2019 [32]	A pulsed THz imaging system with a reflection mode	Paraffin-embedded malignant tissues in human lung and small intestine tissues	The adipose tissue area showed a lower refractive index and with a diffraction-limited spot size of ∼360 *μ*m at 1 THz
Okada et al., 2019 [33]	A scanning laser THz near-field reflection imaging system	Paraffin-embedded human breast tissue	The spatial resolution reached 20 *μ*m
Bowman et al., 2019 [34]	A pulsed THz imaging and spectroscopy system	Freshly excised breast cancer tumors	The cancerous areas exhibited higher absorption coefficients and refractive indexes than normal tissues, and the resolution reached 200 *μ*m

**Figure 2:**
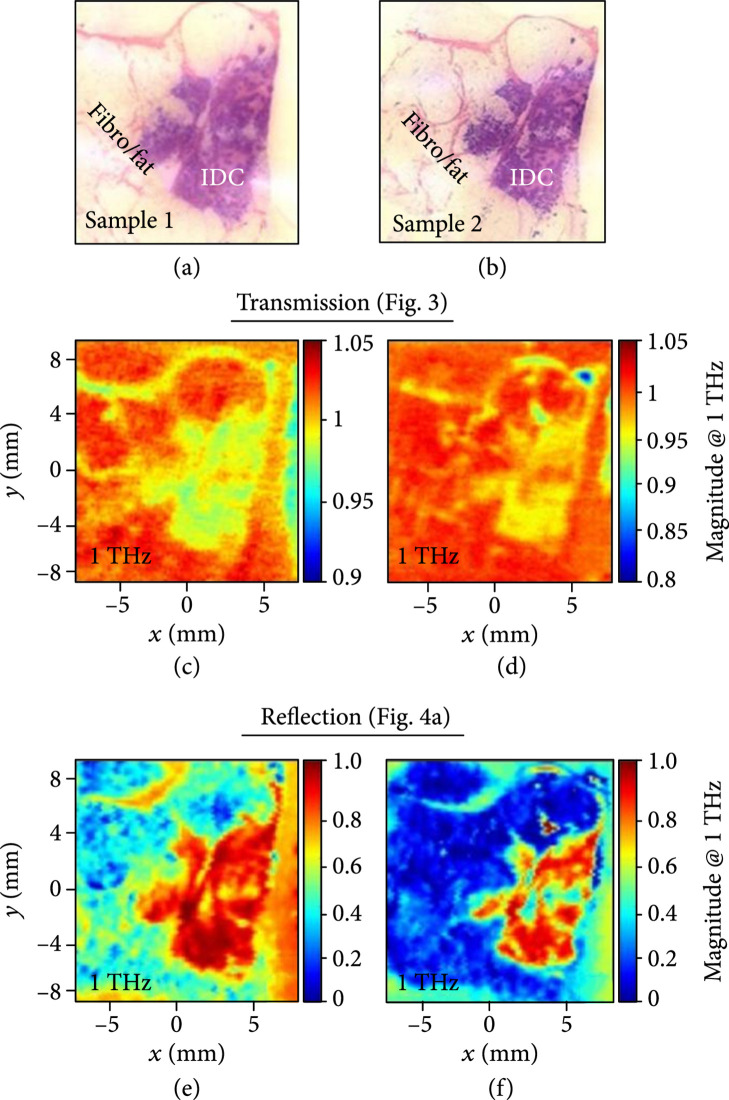
Frequency-domain images of infiltrating ductal carcinoma. Pathology for (a) Sample 1 and (b) Sample 2. Transmission magnitude images at 1 THz of (c) Sample 1 and (d) Sample 2. Reflection magnitude images at 1 THz of (e) Sample 1 and (f) Sample 2 (from Ref. [25]).

Figure 2 shows frequency-domain images of an infiltrating ductal carcinoma at 1 THz from Bowman et al. (2016), where cancerous areas exhibited lower transmission and higher reflection compared with the adjacent normal areas of fibrous and fatty tissues [25]. Similar observations were made in many other studies. The observed contrast was attributed to higher water content in the cancerous areas, which resulted in higher absorption, reflection, or refractive index with THz waves.

**Figure 3:**
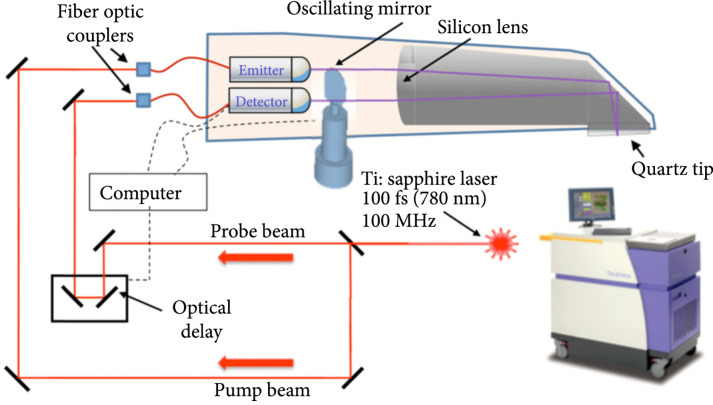
Schematic illustration of the handheld TPI probe system (from Ref. [28]).

For application in rapid cancer screening during surgery, imaging systems must be compact and easy to use. The handheld THz pulsed imaging (TPI) system designed by Grootendorst et al. (2017) shown in the schematic illustration in Figure 3 is a good example of such a system [28]. In this TPI system, the quartz tip scans an area of 15×2 mm, and measurements are collected in 26 pixels. Using this system, the identification accuracy for cancerous areas reached 75%. However, one limitation of this *ex vivo* study is that the sample needs to be sliced into sections of uniform thickness (often on the order of micrometers) to ensure that THz waves can penetrate them.

As the human body contains various components, including large amounts of water, THz waves cannot penetrate deeply. Therefore, most studies rely on *ex vivo* tissue imaging and few have used *in vivo* tissue imaging.

In the past 5 years, only two studies have successfully obtained *in vivo* images by using a pulsed THz reflectometric imaging system. In 2016, Ji et al. obtained images of gliomas from live tumor model mice with a resolution of 250 *μ*m. In these images, the tumor tissue exhibited a higher intensity signal than the adjacent normal tissue [35]. In 2019, Wu et al. also obtained images of brain gliomas in model mice with a resolution of 200 *μ*m by using a THz reflection imaging system with 2.52 THz continuous waves [36]. Currently, *in vivo* THz imaging research is still in the beginning stage. For studies in the human body, two critical issues remain. First, human tissue contains significantly more types of components compared with mouse tissue, which complicated spectral analysis. Second, individual differences in tissue contents will result in differences in absorption and refraction of THz waves between subjects. These two issues seriously interfere with the accurate identification of cancerous areas.

Currently, THz images of cancer tissue are mostly constructed based on differences between the water contents of normal tissue and cancer tissue. Although this can elucidate differences between the cancer tissue and the adjacent area, which gives an intuitive understanding of the cancer tissue, it cannot be used to diagnose the specific cancer type.

## 3. Terahertz Spectroscopy in Cancer Diagnosis

Most biomolecules exhibit unique resonance (*i.e.*, spectral “fingerprints”) in the presence of THz waves. Thus, THz spectroscopy can be used to identify many cancer biomarkers and, therefore, determine the type of cancer. However, in most cases, the biomarker concentration in cancer tissues is small, and the tissue contains hundreds of other substances [37], which makes it difficult to identify the absorption peaks of a biomarker in mixed spectra. Therefore, most current studies examine the biomarkers in their pure state or in simple mixtures (*e.g.*, with ten different substances). To realize cancer detection basing on the spectral fingerprints of biomarkers, spectroscopy systems need to be further developed to achieve higher signal intensity and sensitivity.

### 3.1. Identification of Biomarkers in Cancer

To study the biomarkers in cancer tissue, the first step should be to study the biomarkers themselves. Many research groups have identified the characteristic peaks of biomarkers for different diseases. However, the complicated molecular structures of cancer-related DNA and proteins cannot yet be identified using THz spectroscopy. Smaller molecules are easier to identify and, therefore, have low identification errors; thus, they are the preferred targets for study. *γ*-Aminobutyric acid exists in the central nervous system in various animal species, and its concentration markedly decreases in cancer tissue [38]. Cheng et al. examined the spectrum of *γ*-aminobutyric acid from 0.5 to 18 THz and found absorption peaks at 1.13, 1.52, 2.03, 2.58, 3.48, 4.34, 5.53, 7.80, 8.26, 9.63, 12.0, and 16.7 THz [39]. The neuronal loss caused by tumors also leads to a decrease in N-acetyl-aspartate [40], which exhibits absorption peaks at 1.466, 1.695, 1.979, and 2.879 THz. Chen et al. also reported the THz spectra of the glioma biomarker 2-hydroxyglutaric acid disodium salt (2HG), which exists as two different isomers with different spectra: L-2HG exhibits characteristic peaks at 0.769, 1.337, 1.456, and 1.933 THz, while D-2HG exhibits characteristic peaks at 0.760, 1. 200, 1.695, and 2.217 THz. Therefore, THz spectroscopy can be used to accurately and rapidly identify both isomers of 2HG, so it may be used as a tool for the early diagnosis of glioma [41]. Yang et al. measured the THz spectrum of myo-inositol, which is found in elevated levels in gliomatosis [42], and reported that it exhibits four characteristic peaks at 1.00, 1.46, 1.58, 1.85, and 2.05 THz [43].

These findings showing the resonance absorption peaks of several cancer-related biomolecules in the THz range demonstrate that THz spectroscopy can be a potential tool for biomarker detection in cancer diagnosis. However, because the biomarkers are found in low concentrations and obscured by hundreds of other substances that are also present in the tissue, the accurate THz-based detection of biomarkers is extremely difficult using existing technologies.

### 3.2. THz Spectral Algorithms for Cancer Diagnosis

Because the changes in biomarker concentration intensify with the development of cancer, the quantitative analysis of biomarker concentrations can help with cancer staging [44]. Various regression algorithms have been used to realize quantitative biomarker analysis. However, as mentioned before, it is currently difficult to identify biomarkers in tissue samples. Therefore, most of these preliminary component analysis studies used engineered samples with no more than ten substances.

**Figure 4:**
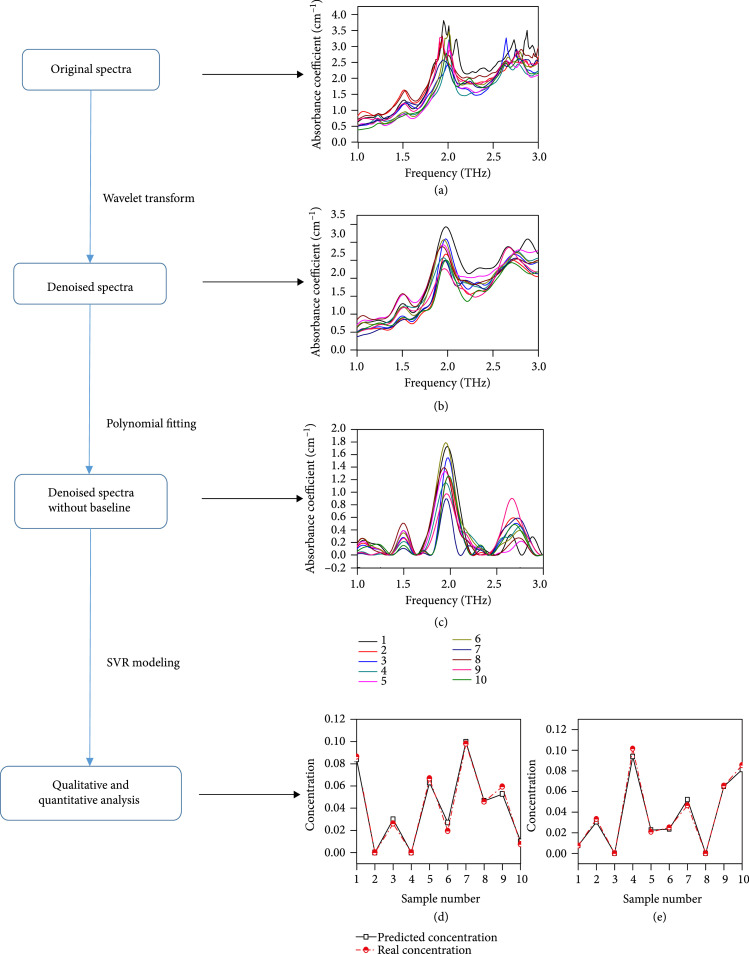
Diagram of the proposed analysis algorithm. (a) The original spectra of ten samples with seven components in different proportions. (b) The denoised spectra of samples processed by wavelet transform. (c) The denoised spectra without baseline after processing by polynomial fitting. (d) Actual and predicted concentrations of N-acetylaspartate. (e) Actual and predicted concentrations of noradrenaline (from Ref. [47]).

In 2016, Ge et al. constructed linear and nonlinear models to quantitatively analyze the concentration of aflatoxin B1, which is a carcinogen. They combined THz spectroscopy with the partial least squares (PLS), principal component regression (PCR), support vector machine (SVM), and PCA-SVM methods. The results showed that the PLS and PCR models provided the best aflatoxin B1 quantification results in the concentration range of 1-50 *μ*g/mL; the accuracy was as high as 87.5%. In the concentration range of 1-50 ng/mL, SVM and PCA-SVM showed the best results, and the accuracy reached 93.75% [45]. Lu et al. qualitatively and quantitatively analyzed binary amino acid mixtures using PLS and interval partial least square (iPLS) regressions. The iPLS regression resulted in a lower root mean square error (0.39%) for the two components than the PLS method [46]. In 2018, Peng et al. reported a method to analyze the main substances in human brain tissue cells, including *γ*-aminobutyric acid, L-glutamic acid, D-myo-inositol, creatine monohydrate, cholesterol, noradrenaline, and N-acetylaspartate. When cancer occurs, the concentrations of noradrenaline and N-acetylaspartate will increase and decrease, respectively. A diagram of the proposed algorithm is shown in Figure 4. The algorithm comprises three steps: wavelet transform for denoising, polynomial fitting for to remove the baseline signal, and support vector regression (SVR) modeling for analysis. This approach was shown to predict the concentration of these two substances among all seven substances with a root mean square error of 0.40% [47].

Algorithms have been also used to differentiate cancerous tissue from normal tissue based on their spectral differences. A few studies have succeeded in identifying cancer in real tissue samples rather than engineered mixtures. In 2015, Qi et al. combined THz spectroscopy with a fuzzy rule-building expert system (FuRES) and fuzzy optimal associative memory (FOAM) for the diagnosis of cervical carcinoma. In a test on 52 cervical tissue sections (32 normal and 20 cancerous), the classification accuracy of the FuRES reached 92.9%±0.4%, and that of FOAM reached 92.5%±0.4% [48]. In 2018, Liu et al. combined THz spectroscopy with two linear compression methods (principal component analysis and locality preserving projections (LPP)) and a nonlinear method (Isomap) for the identification of hepatic tumors. The best classification accuracies from two-dimensional time-domain data were achieved with an Isomap probabilistic neural network (PNN; 99.81%±0.30%) and an Isomap support vector machine (SVM; 99.69%±0.61%). The best classification results from two-dimensional frequency-domain data were achieved by LPP-PNN (100.00%±0.00%) and LPP-SVM (99.75%±0.32%) [49]. In 2020, Liu et al. proposed a method of wavelet entropy feature extraction and a machine learning classifier for the recognition of breast invasive ductal carcinoma from THz pulsed signals. Results showed that cancer identification was achieved with a precision of 92.85% [50].

These studies demonstrated the feasibility of using various algorithms in combination with THz spectroscopy to diagnose cancer. However, the key remaining issue is that the identification accuracy will decrease with increasing numbers of components in the mixture. Therefore, the algorithms for qualitative analysis of biomarkers are still not applicable for tissue samples. To solve this problem, some groups proposed different methods.

## 4. Methods of SNR Enhancement in THz Measurements for Cancer Diagnosis

As mentioned above, one of the critical issues associated with THz detection of biomarkers for cancer diagnosis is that tissue strongly absorbs THz waves, which leads to a poor SNR in the obtained spectra. Therefore, several research groups have used different methods to enhance the SNR of THz spectra.

Some groups have used contrast agents to enhance the contrast of cancerous areas. In 2016, Zhang et al. proposed the use of superparamagnetic iron oxide nanoparticles (SPIOs) to increase the reflection of THz waves in water upon exposure to an alternating magnetic field. Focal-plane imaging experiments were conducted on water with and without 4 g/L SPIOs, and the average amplitude of the relative reflection was 29.41%±0.42% in water with SPIOs compared with only 0.30%±0.03% in water without SPIOs. As the imaging contrast between cancerous and normal tissues depends on the difference in the water contents in these tissues, the SPIOs can enhance the contrast of cancerous areas relative to normal areas [51]. In 2017, Bowman et al. proposed the use of nanometer-scale onion-like carbon (OLC) that can be activated for selective binding to cancer cells to increase the reflection of cancerous tissues. Experimental results showed that an invasive ductal carcinoma phantom with 10% OLC exhibited drastically higher reflection compared with the surrounding fibroglandular (healthy) tissues [52]. In 2019, Huang et al. designed silica-coated gold nanorods as a contrast agent for imaging analyzing prostate cancer cells. The intensity of the cancerous area with silica-coated gold nanorods was 25.35% higher than that of the sample without nanoparticles [53].

Optical clearing agents have been used to reduce the water inside the issue for THz spectroscopy, reducing the absorption of THz waves to improve the spectral SNR and allow for easier identification of biomarkers. In 2018, Musina et al. proposed several THz wave penetration-enhancing agents for optical clearing of tissues: polyethylene glycol with different molecular weights, propylene glycol, ethylene glycol, and dimethyl sulfoxide. These agents have about three times lower absorption than water [54]. Yang et al. used fluorinated oil as an optical clearing agent to replace the liquid medium around living cells. An independent t-test revealed that the difference between the absorption of fluorinated oil with and without cells was statistically significant (P<0.05 at 0.5, 1.0, and 1.5 THz) [55]. These studies demonstrated that optical clearing agents can be used to reduce the noise from water absorption, which enhances the spectral SNR and allows biomarkers to be identified more effectively.

Antibody-based biosensors can be used for the detection of cancer cells. Figure 5 shows a biosensor designed by Hassan et al. that comprises a silicon dioxide layer coated with aptamers [56]. The biosensor was used to detect metastatic breast cancer cells as follows: the antibody binds only to cancer-related cell surface protein, changing the THz absorption of these cancer cells relative to normal cells. The experimental results showed that the THz amplitude changed linear change with the cancer stage. The detection limit was one breast cancer cell in a 100 *μ*L volume. This approach is a good method for detecting biomarkers that do not have characteristic absorption peaks in the THz band. However, the amplitude of a THz signal may be influenced by the environment (such as humidity), which may cause jitter in the signal and lead to error.

Figure 5Schematic diagram of the experimental design of an antibody-based biosensor. (a) Modification of the SiO_2_ layer to immobilize MAMB1 and MAMA2 aptamers. (b) After immobilization, different numbers of cells were added to each sensing plate, and the THz amplitude was recorded (from Ref. [56]).

**(a):**
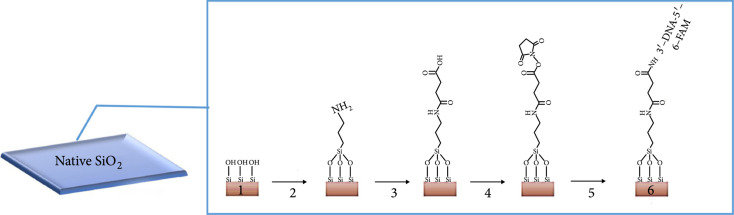

**(b):**
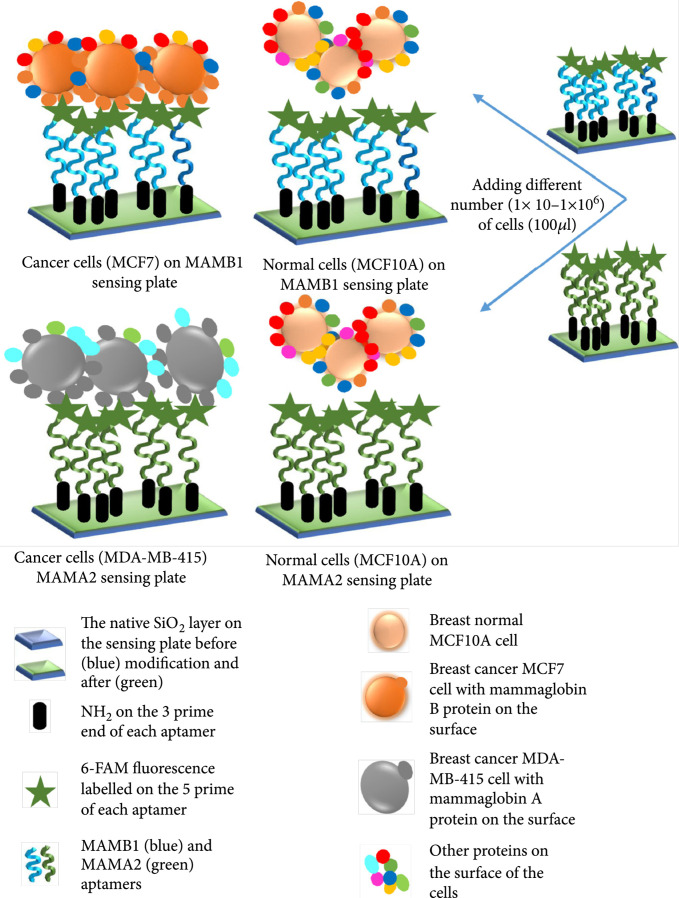

To minimize the error and further enhance the spectral SNR, metamaterials have been designed as biosensors. These metamaterials exhibit resonance peaks with high spectral SNR in the THz range. The peaks exhibit frequency shifts according to the refractive index of the sample that the metamaterial is in contact with. Thus, these metamaterials can be used in combination with antibodies to detect cancer biomarkers or cells. In 2016, Geng et al. demonstrated metamaterials with specific antibodies to detect two biomarkers of liver cancer: alphafetoprotein and glutamine transferase isozyme II. This biosensor exhibited detection limits of 5 mu/mL and 0.02524 *μ*g/mL for alphafetoprotein and glutamine transferase isozyme II, respectively [57]. In 2018, Liu et al. developed a bow-tie THz metamaterial biosensor coated with an antibody for the epidermal growth factor receptor (EGFR), which is a biomarker for gastrointestinal cancer, breast cancer, head-and-neck cancer, and epithelial cancer. This biosensor exhibited a detection limit of 10 fmol/mL [58]. In 2020, Weisenstein et al. designed a metamaterial biosensor combined with complementary DNA strands that bind specifically to single-stranded oligo- or poly-nucleotide probe molecules. This biosensor was used for the detection of a human tumor marker, melanoma inhibitory activity (MIA) mRNA, with a detection limit of $1.55\times 10^{-12}\;\text{mol}/\text{L}$ [59].

These proposed auxiliary methods all succeeded in enhancing the spectral SNR. Contrast agents, optical cleaning agents, antibodies, and metamaterials can specifically elevate or eliminate the signals of certain substances, thus enhancing the final spectral SNR. However, sample contamination with additional substances is unavoidable. Furthermore, substances for *in vivo* use must not cause harm to the human body. Antibodies and metamaterials can be used in combination to enhance the detection sensitivity and identify biomarkers in low concentrations. However, the process of searching for antibodies is not easy, and there are no antibodies available for some cancer biomarkers.

## 5. Conclusion

In this article, we reviewed many studies conducted in the past 5 years that focus on THz imaging and THz spectroscopy applied in cancer diagnosis. THz imaging can clearly differentiate cancerous tissue from normal tissue with clear boundaries based on the difference in the water contents of these tissues. However, because of the high absorption of THz waves by water and other substances present in tissue, the penetration depth of THz waves is limited to 1 mm. Therefore, current THz imaging studies in oncology focus on excised tissue or use reflection imaging systems to study the surface layer of the tissue. Furthermore, THz imaging based only on water content cannot be used to determine the type of cancer.

Biomarkers can be used for cancer identification in most cases. Some biomarkers exhibit resonance with THz waves, so THz spectroscopy can be a tool for biomarker identification in cancer tissue. However, the low concentrations of biomarkers and the presence of various outer substances in the cancer tissue make it difficult to identify biomarkers in real tissues. Therefore, current THz spectroscopy technologies can only identify biomarkers in engineered mixtures with no more than ten substances.

Auxiliary methods have been developed to address these issues. These methods included the use of contrast agents, optical cleaning agents, antibodies, and metamaterials to enhance the spectral SNR. However, sample contamination by additional substances is unavoidable. Antibodies have been combined with metamaterial biosensors to achieve highly sensitive biomarker detection. However, the process of developing antibodies for biomarkers is complicated, and antibodies may not be available for some cancer biomarkers.

In summary, the studies presented here have demonstrated the feasibility of using THz technologies in cancer diagnosis. However, additional development is needed before these technologies can be practically applied for the early diagnosis of cancer. Future developments of THz technologies for cancer diagnosis include combining THz imaging with THz spectral fingerprinting of biomarkers to realize qualitative identification and quantitative analysis simultaneously. To achieve this objective, further improvements in THz systems and auxiliary methods are needed.

## Data Availability

The datasets used and/or analyzed during the current study are available from the corresponding author on reasonable request.
